# 797. Investigation of HIV Clusters Among Hispanic/Latino Gay or Bisexual Men in Metro Atlanta, Georgia

**DOI:** 10.1093/ofid/ofac492.057

**Published:** 2022-12-15

**Authors:** Carlos S Saldana, Rebecca Hershow, David Philpott, Rashida Hassan, Kathryn Curran, Jenna Gettings, Eleanor Garlow, Daniel Mauck, Valeria D Cantos, David P Holland, Dorian Freeman, Erica Johnson, Karrie Reed, Jose Adame, Humberto Orozco, Pascale Wortley

**Affiliations:** Emory University School of Medicine, Atlanta, Georgia; Centers for Disease Control and Prevention, Atlanta, Georgia; Centers for Disease Control and Prevention, Atlanta, Georgia; Centers for Disease Control and Prevention, Atlanta, Georgia; Centers for Disease Control and Prevention, Atlanta, Georgia; Centers for Disease Control and Prevention, Atlanta, Georgia; Georgia Department of Public Health, Atlanta, Georgia; Georgia Department of Public Health, Atlanta, Georgia; Emory University School of Medicine, Atlanta, Georgia; Emory University School of Medicine, Atlanta, Georgia; Gwinnett, Newton & Rockdale County Health Departments, Lawrenceville, Georgia; DeKalb County Board of Health, Decatur, Georgia; Cobb & Douglas Public Health, Marrietta, Georgia; Georgia Department of Public Health, Atlanta, Georgia; Latino LinQ, Atlanta, Georgia; Georgia Department of Public Health, Atlanta, Georgia

## Abstract

**Background:**

In Metro Atlanta, Georgia, annual HIV diagnoses among Hispanic/Latino (H/L) adolescents and adults have increased since 2014 **(**Figure 1**),** and four HIV molecular clusters, consisting primarily of Hispanic/Latino gay or bisexual men and other men who have sex with men (HLGBM), were identified in 2021. In March 2022, the Georgia Department of Public Health, the Centers for Disease Control and Prevention, and four health districts of Metro Atlanta (Fulton, Gwinnett, DeKalb, and Cobb) launched an investigation to characterize the clusters, assess barriers to accessing HIV care and prevention services, and inform improvements to service delivery.

**Methods:**

We described the four clusters using HIV surveillance data. We conducted semi-structured qualitative interviews with 29 HLGBM and 28 providers through purposive sampling. Iterative analyses were conducted daily, comparing findings across interviews to identify commonly mentioned barriers to accessing HIV care and prevention services.

**Results:**

The four clusters varied in size (5–42 members) and proportion of H/L members (41–100%); one cluster included members reporting injection drug use (14%) (Table 1). Viral suppression among cluster members was high across clusters (87–100%). Overarching reported barriers to accessing medical services included lack of culturally and linguistically concordant services, fear of deportation, transportation, and financial barriers (Table 2). LGBTQ and HIV stigma, low STD/HIV awareness, low access to primary care and HIV screening in primary and urgent care settings, and limited community outreach and marketing were common barriers to accessing HIV prevention services. For pre-exposure prophylaxis (PrEP) specifically, fear of being perceived as promiscuous, limited PrEP knowledge, and concerns with side effects were barriers. Navigating the healthcare system was noted as the primary challenge in HIV care.

**Conclusion:**

The investigation of four clusters affecting HLGBM identified opportunities to improve access to HIV prevention and PrEP services for this population. State and local partners are planning cluster response activities, including ways to provide low-barrier culturally and linguistically concordant services **(**Table 3**).**

**Disclosures:**

**All Authors**: No reported disclosures.

